# Supplementary material for: Chromenopyrimidinone Controls Stemness and Malignancy by suppressing CD133 Expression in Hepatocellular Carcinoma
Source: Cancers (Basel). 2020 May 8;12(5):1193. doi: 10.3390/cancers12051193 (PMC7281429; doi:10.3390/cancers12051193)
Supplement: Supplementary file 1 [file cancers-12-01193-s001.pdf]

Supplementary Material: Chromenopyrimidinone Controls Stemness and Malignancy by suppressing CD133 Expression in Hepatocellular Carcinoma

Yeonhwa Song, Sanghwa Kim, Hyeryon Lee, Joo Hwan No, Hyung Chul Ryu, Jason Kim, Jee Woong Lim, Moonhee Kim and Inhee Choi and Haeng Ran Seo

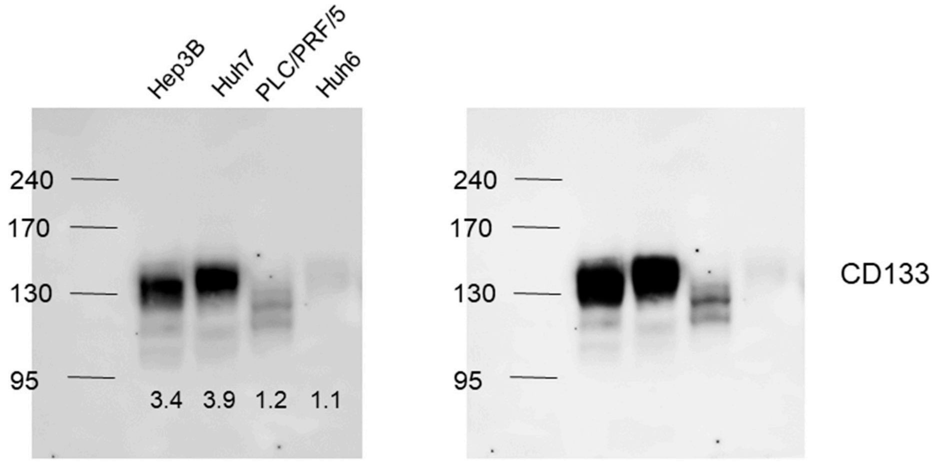

Figure S1. Detailed information about Figure 1E.

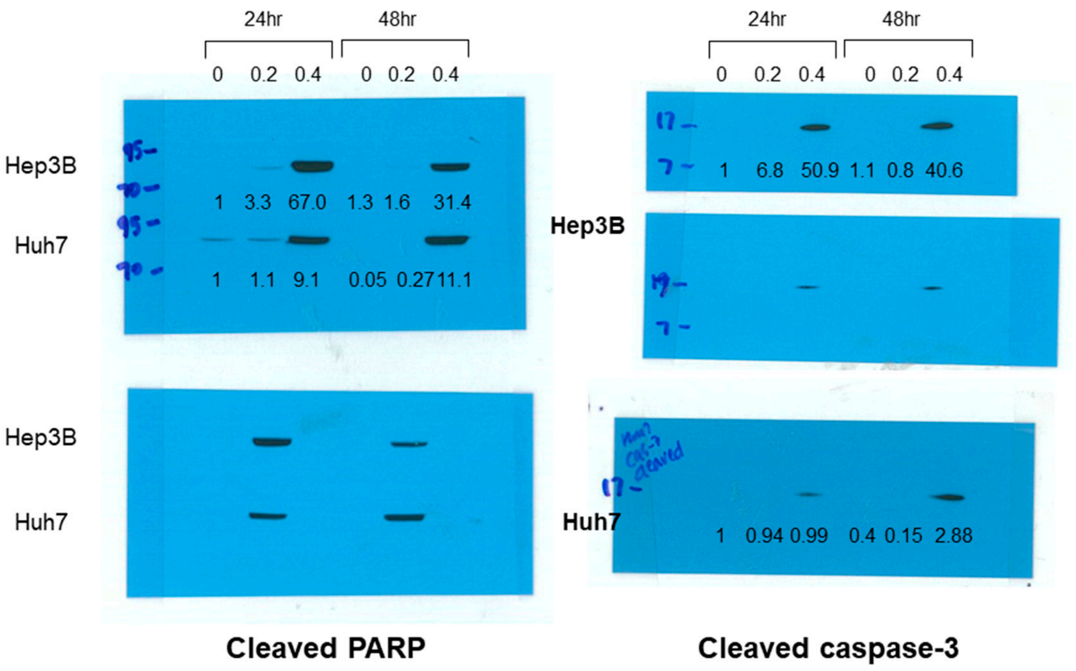

Figure S2. Detailed information about Figure 2D.

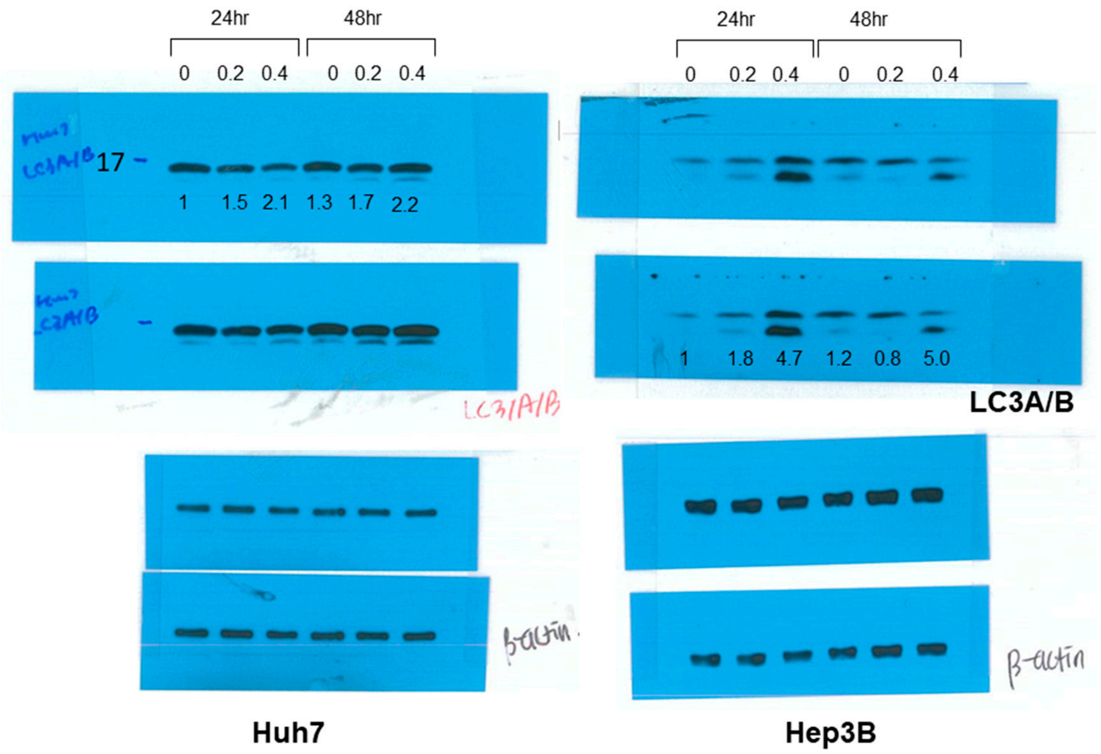

Figure S3. Detailed information about Figure 3C.

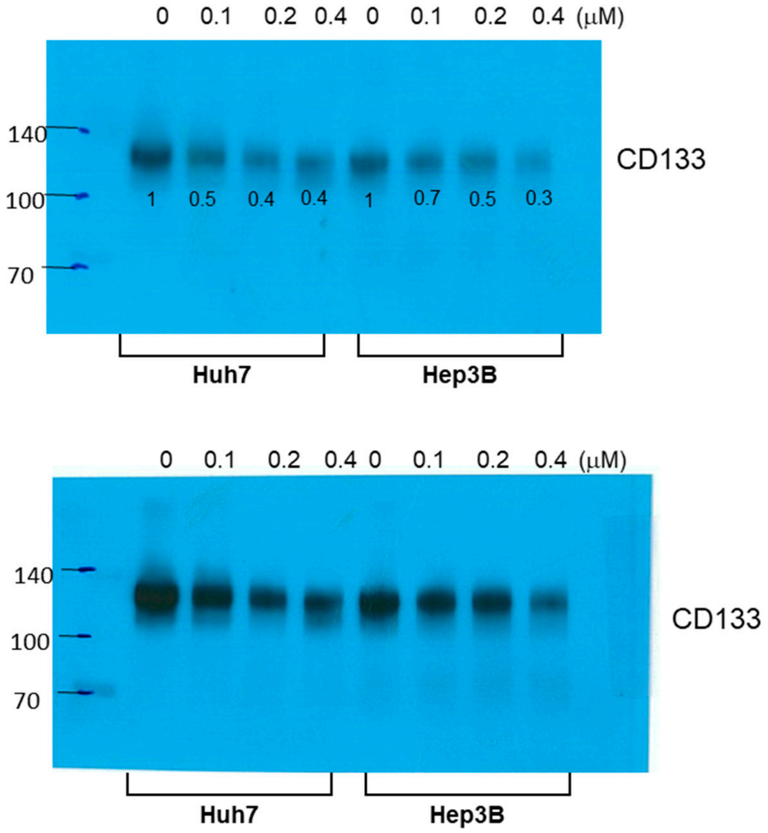

Figure S4. Detailed information about Figure 4B.

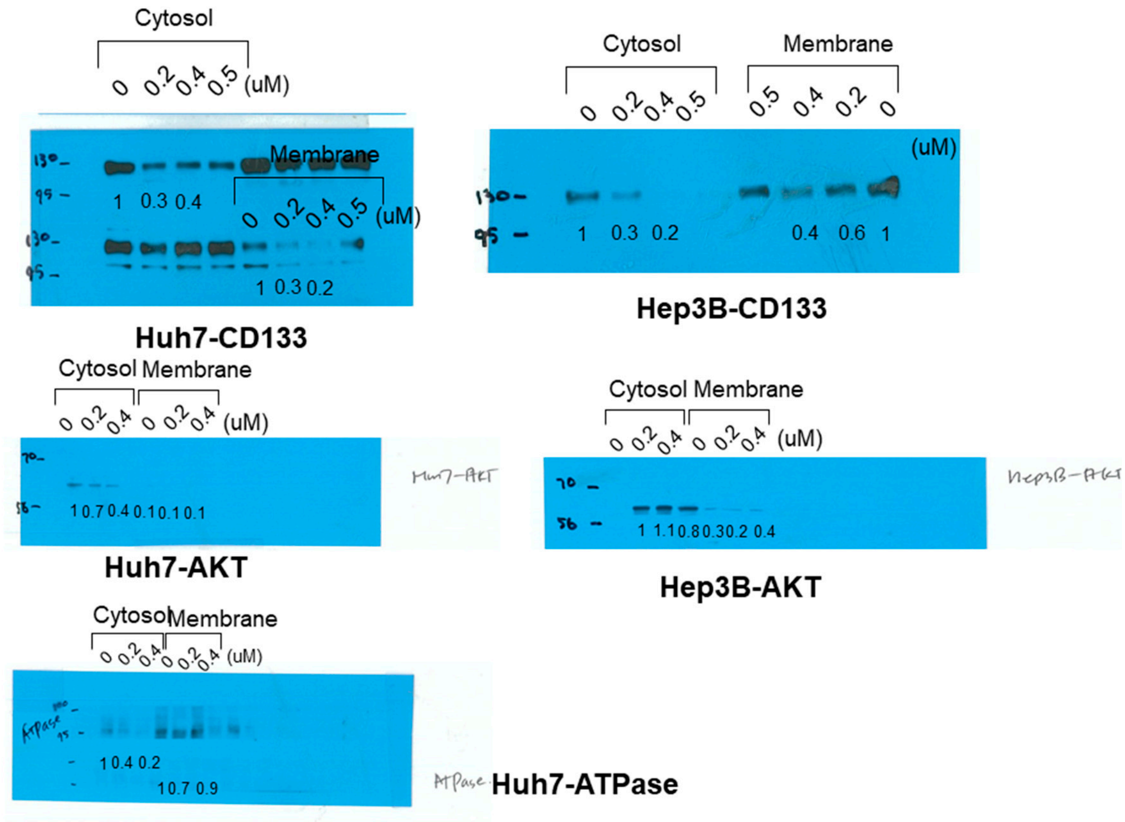

Figure S5. Detailed information about Figure 4D.

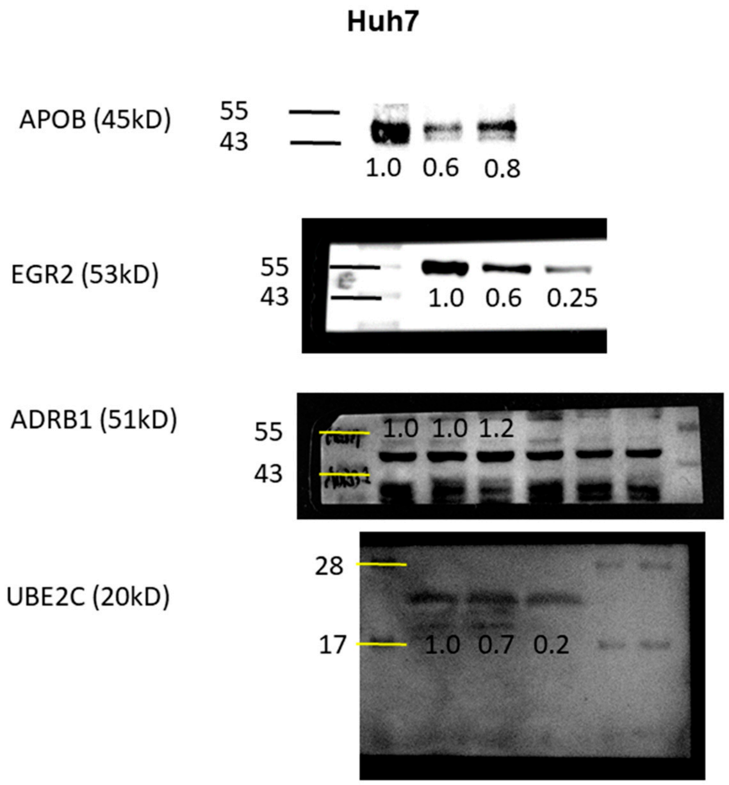

Figure S6. Detailed information about Figure 6C (Huh7).

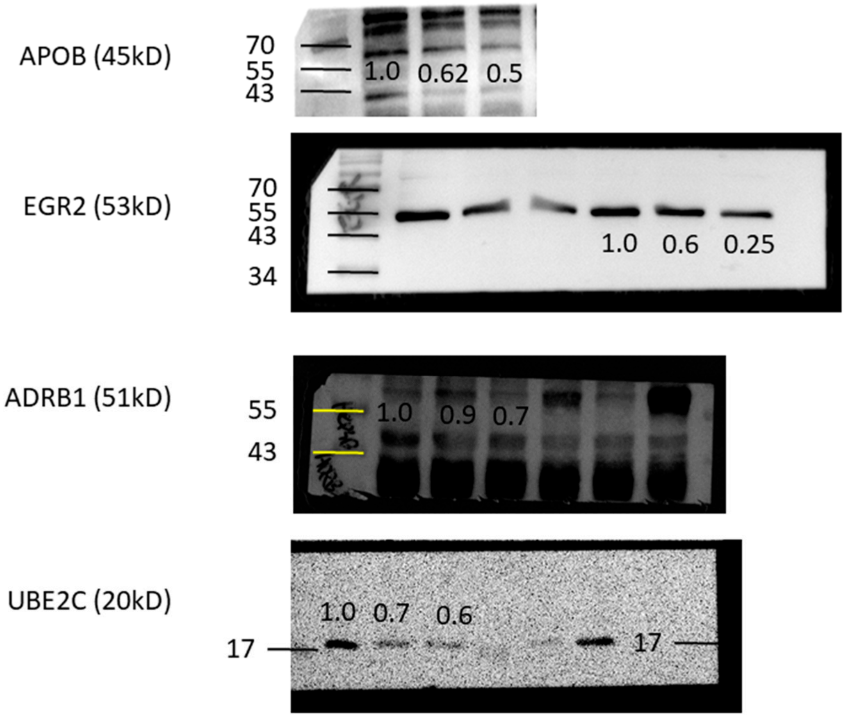

Figure S7. Detailed information about Figure 6C (Hep3B).

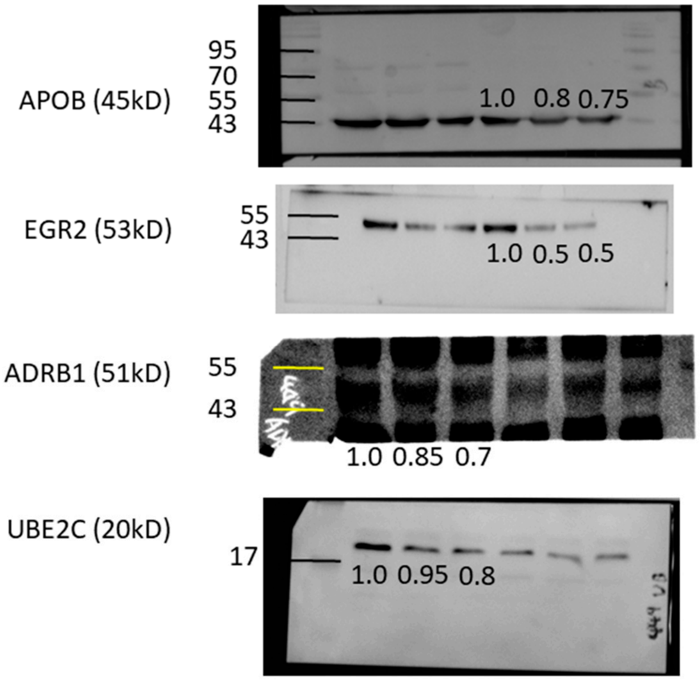

Figure S8. Detailed information about Figure 6C (SNU449).

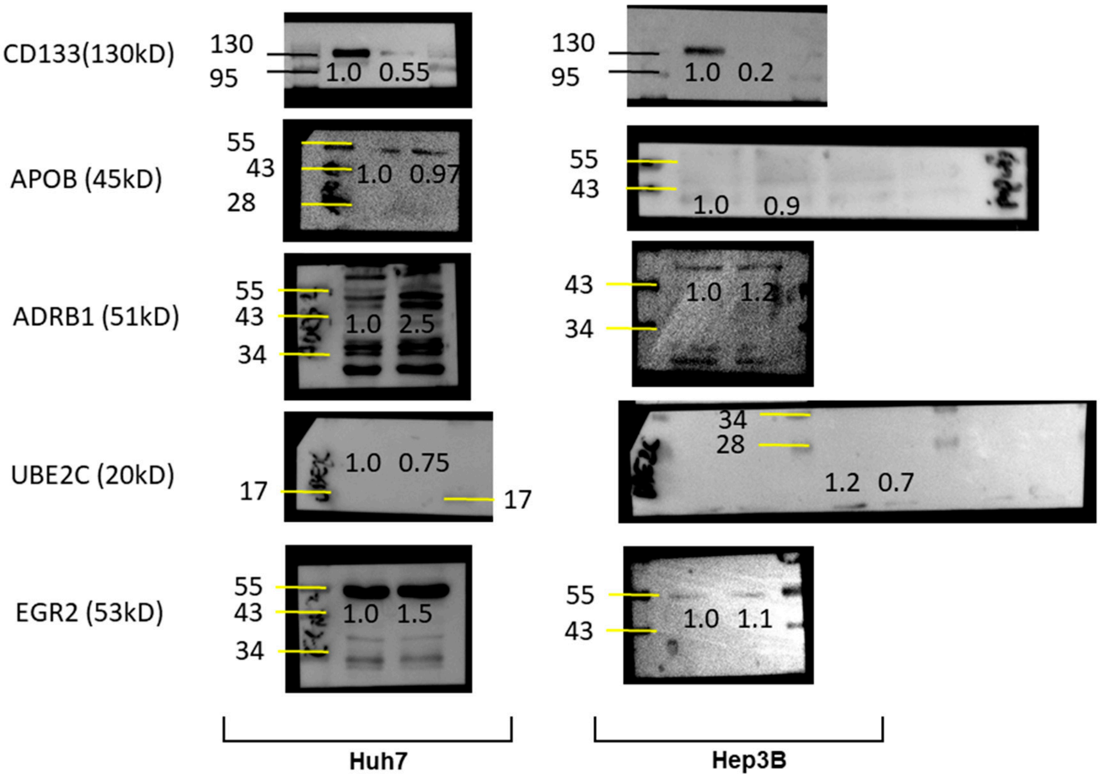

Figure S9. Detailed information about figure 6D.
